# Clinical presentation of Warburg effect in aggressive lymphoma: a case report

**DOI:** 10.1186/s13256-023-04079-6

**Published:** 2023-08-23

**Authors:** Yenong Cao, Margaret C. Liu, Emma L. Hanlon, York Chen, Muhammad Z. Afzal, Christi A. Hayes, John M. Hill

**Affiliations:** 1 Department of Hematology and Oncology, Tufts Medical Center, 800 Washington Street, Boston, MA 02111 USA; 2 Division of Gastroenterology and Hepatology, Mayo Clinic, 13400 E Shea Blvd, Scottsdale, AZ 85259 USA; 3 Beth Israel Deaconess Medical Center, 330 Brookline Ave, Boston, MA 02215 USA; 4 Department of Internal Medicine, Dartmouth Health, 1 Medical Center Drive, Lebanon, NH 03756 USA; 5 Division of Hematology and Oncology, Dartmouth Cancer Center, 1 Medical Center Drive, Lebanon, NH 03756 USA

**Keywords:** Warburg effect, Diffuse large B cell lymphoma, Acute renal failure, Case report

## Abstract

**Background:**

The Warburg effect is a rare condition in tumor biology, illustrated by significant lactate production in the presence of oxygen. The Warburg effect is associated with very poor prognosis in patients with malignancy.

**Case presentation:**

We report a 76-year-old Caucasian woman with double-expressor diffuse large B cell lymphoma who presented with severe lactic acidosis and extreme hypoglycemia with normal mentation. Her lactic acidosis was initially controlled with a bicarbonate infusion, and the patient was started promptly on steroids, followed by chemotherapy, but her clinical course was complicated by tumor lysis syndrome, acute renal failure requiring hemodialysis, and progressive liver failure. She manifested a temporary clinical response to chemotherapy but eventually died of complications.

**Conclusions:**

This case demonstrates the importance of prompt recognition of the Warburg effect, aggressive supportive measures, and early initiation of chemotherapy. Future studies are needed to characterize the role of hemodialysis in this setting.

## Introduction

The Warburg effect was first described by Otto Warburg in the 1920s and is characterized by increased glucose uptake leading to extreme lactate production by tumors in the presence of oxygen [1]. Since lactate is typically produced by glycolysis in an anaerobic environment, the Warburg effect is also known as aerobic glycolysis. As a subtype of Type B lactic acidosis, the Warburg effect only rarely occurs in patients with leukemia, lymphoma, or solid malignancies [2].

Thus, few cases of the Warburg effect with lactic acidosis and hypoglycemia in lymphoma have been reported, and these have been associated with a very grim prognosis, leading to 70–80% mortality in the first month following diagnosis [3]. The definitive treatment for malignancy-associated lactic acidosis is chemotherapy.We report a case of a patient with double-expressor diffuse large B cell lymphoma (DLBCL) who presented with severe lactic acidosis and hypoglycemia complicated by acute renal failure requiring dialysis.

## Case presentation

**Fig. 1:**
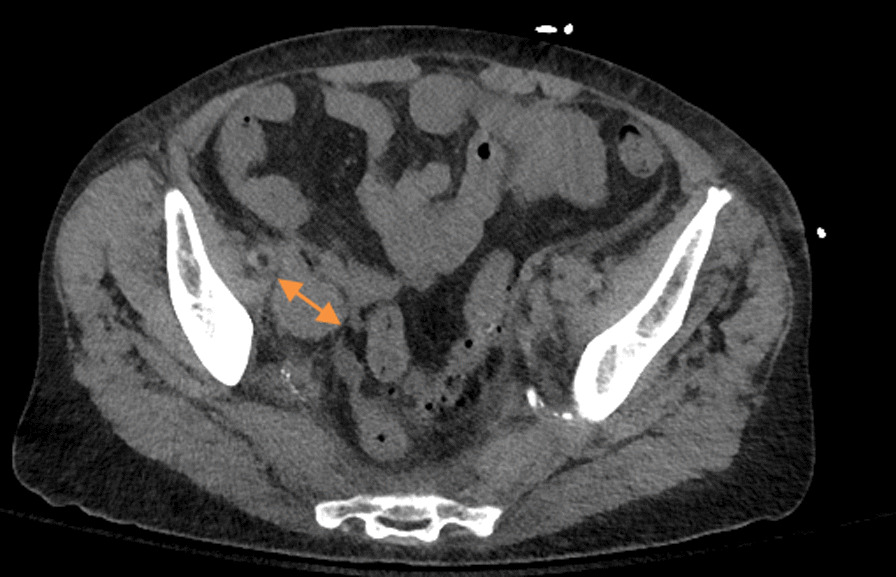
CT abdomen/pelvis without contrast. Orange marker demonstrates the largest retroperitoneal lymphadenopathy, measured at 24.6 mm

A 76-year-old Caucasian woman with no significant past medical history and incomplete primary care follow-up presented to an outside hospital with several months of fatigue, generalized weakness, lethargy, and > 10% unintentional weight loss. Her admission labs were significant for white blood cell count of 24.2 × 10^3^/mcL (4.0–10.0 × 10^3^/mcL), hemoglobin 12.6 g/dL (12.0–15.0 g/dL), platelets 119 × 10^3^/mcL (150–450 × 10^3^/mcL), potassium 5.6 mmol/L (3.5–5.0 mmol/L), CO_2_ 7 mmol/L (23–29 mmol/L),blood urea nitrogen (BUN) 55 mg/dL (6–20 mg/dL), creatinine 1.63 mg/dL (0.80–1.20 mg/dL), glucose 9 mg/dL (64–100 mg/dL), alkaline phosphatase 202 units/L (20–130 units/L), aspartate aminotransferase (AST) 273 units/L (8–33 units/L), alanine transaminase (ALT) 75 units/L (4–36 units/L), total bilirubin 2.1 mg/dL (0.1–1.2 mg/dL) (direct bilirubin 1.7 mg/dL), albumin 3.1 mg/dL (3.4–5.4 mg/dL), pH 6.93 (7.35–7.45), and lactate 19 units/L (0–2 units/L). She was started on a 10% dextrose (D10) infusion with 150 mEq of sodium bicarbonate, and notably would become hypoglycemic whenever it was attempted to titrate her off the D10 infusion. Her acidosis was initially responsive to the sodium bicarbonate infusion with her pH correcting to 7.46 prior to admission to our facility. A computed tomography (CT) scan of the chest, abdomen, and pelvis was notable for pathologic retroperitoneal, pelvic, and mediastinal lymphadenopathy (Fig. 1). A CT-guided retroperitoneal lymph node biopsy was performed, and preliminary results showed large cell lymphoma of likely B-cell origin, though flowcytometry was unremarkable. This prompted her transfer for consideration of emergent chemotherapy.

Upon admission to our facility, despite profound metabolic abnormalities, her mentation was intact. Due to the severity of her metabolic abnormalities, she was immediately started on high-dose prednisone (100 mg daily), and definitive chemotherapy was deferred while awaiting the formal pathology results. She also received one dose of rasburicase 3 mg intravenous for tumor lysis syndrome, as uric acid was 9 mg/dL (3.5–7.2 mg/dL). For her lactic acidosis, she was initially maintained on aggressive fluid repletion and a sodium bicarbonate infusion. This improved her pH to 7.08, though her lactate persisted at 21 units/L. For profound hypoglycemia, she was continued on a D10 infusion. As her lab abnormalities persisted and the diagnosis of DLBCL was confirmed, she was started on standard R-CHOP induction therapy: rituximab (375 mg/m^2^), cyclophosphamide (750 mg/m^2^) on day 1, doxorubicin (50 mg/m^2^) on day 1, vincristine (1.4 mg/m^2^; capped here at 1 mg intravenous pyelogram) on day 1, in conjunction with ongoing prednisone. The final pathology from the lymph node biopsy revealed double-expressor (Bcl2+, MYC+), and other adverse features (non-GCB subtype; MUC1oncoprotein) indicating an aggressive form of DLBCL.

**Fig. 2:**
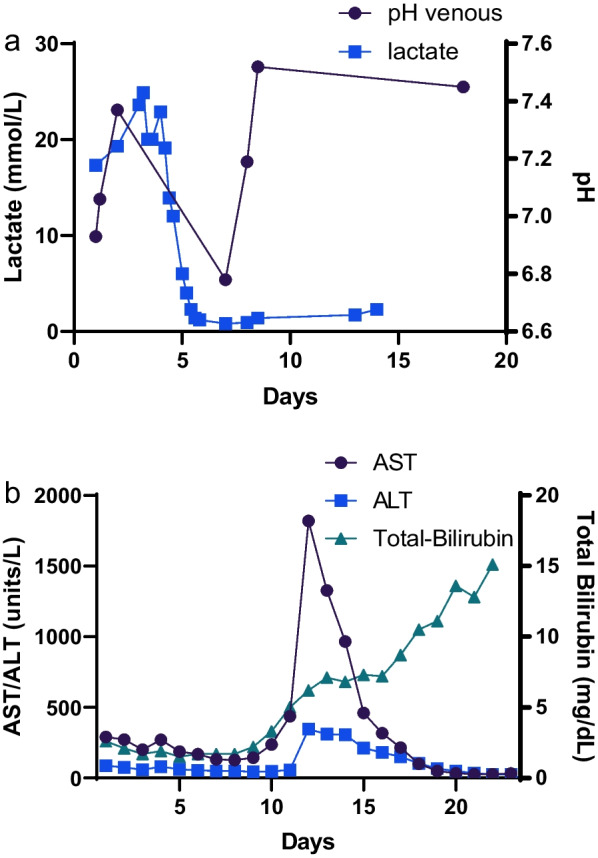
**a **Blood lactate and venous pH levels after the start of chemotherapy (D7) and normalization after initiation of CVVH (D8). **b** Blood AST, ALT, and total bilirubin levels

Despite emergent initiation of R-CHOP, along with continued bicarbonate infusion and fluid repletion, profound lactic acidosis persisted, prompting day 7 transfer to the medical intensive care unit (MICU). Continuous veno-venous hemofiltration (CVVH) was initiated due to oliguria and volume overload with severe metabolic acidosis (pH 6.78 and CO_2_ 2 mmol/L) (Fig. 2a) refractory to medical management. She then became hypotensive and required norepinephrine and vasopressin. While her AST and ALT peaked and then trended downward, the direct hyperbilirubinemia continued to progress, suggesting an ischemic etiology (Fig. 2b). Hepatic ultrasound demonstrated two large liver cysts as well as gallbladder sludge and nonspecific diffuse gallbladder wall thickening without biliary duct dilatation. For presumed septic shock she was treated empirically with piperacillin-tazobactam and vancomycin, also developing disseminated intravascular coagulation (DIC) and requiring cryoprecipitate infusions. Her MICU course was further complicated by a small subarachnoid hemorrhage, stabilized via DIC management and platelet transfusions.

She improved clinically and was transferred back to the hematology service from the MICU after 5 days, with continuation of intermittent hemodialysis. Lactic acidosis resolved on hospital day 8 (Fig. 2a). She developed neutropenia as expected from chemotherapy and was started on levofloxacin prophylaxis. Despite continued rise in direct hyperbilirubinemia (Fig. 2b), a repeat right upper quadrant ultrasound was unchanged. On hospital day 17, she developed hypotension and tachycardia, requiring another MICU transfer 12 days after starting R-CHOP. Her blood cultures times 2 from the day of her second MICU transfer grew vancomycin-resistant *Enterococcus faecium*. A family conference was arranged, and she was transitioned to comfort care. She passed away the following day.

## Discussion

This represents a unique case of aggressive double expressor DLBCL that resulted in profound hypoglycemia and lactic acidosis consistent with the Warburg effect. It is intriguing that this patient’s initial presenting glucose was 9 mg/dL (70–100 mg/dL) with normal mentation. The central nervous system (CNS) requires a steady supply of glucose from peripheral circulation, as the brain itself does not synthesize or store a sufficient amount of glucose. In general, neurologic impairment starts to manifest at a glucose level < 50 mg/dL [4]. However, cases of patients with lactic acidosis and intact mentation despite severe hypoglycemia with glucose less than 30 mg/dL have been reported [5]. The theory of lactate-protected hypoglycemia has been proposed, suggesting that lactate may act as an alternate glucose resource for the CNS, while maintaining intact neurologic function in cases of severe hypoglycemia [6].

Acute liver failure is characterized by transaminitis, hepatic encephalopathy, and prolonged prothrombin time (INR ≥ 1.5) in a patient without cirrhosis or preexisting liver dysfunction. In this case, the patient with unknown baseline liver function presented initially with idiopathic hepatic dysfunction, then acute exacerbation in the setting of subsequent hypotension and pressor requirement. Her AST and ALT peaked and decreased rapidly thereafter, with ongoing rise in direct hyperbilirubinemia, a typical pattern for ischemic hepatitis. To our knowledge, liver dysfunction has not been considered a component of the Warburg effect. Acute liver failure and cirrhosis are known to cause lactic acidosis due to decreased lactate clearance. However, it is unknown to what degree lactic acidosis can contribute to liver dysfunction [7]. It is crucial to maintain the blood pH within a narrow range between 7.35 and 7.45 to sustain normal cellular metabolism. According to historical data, a pH of less than 6.8 or greater than 7.8 is considered incompatible with life. However, multiple case reports have reported patients who survived severe metabolic acidosis with a pH of 6.3–6.4, secondary to metformin-associated lactic acidosis [8]. Timely reversal of the cause and initiation of sodium bicarbonate infusion and CVVH were the keys to survival in these cases.

The initial treatment of lactic acidosis includes a sodium bicarbonate infusion and hemodialysis. A bicarbonate infusion is usually started first due to readily available vascular access. Due to the rarity of lactic acidosis associated with malignancy, the impact of intravenous bicarbonate on mortality in the setting of malignancy-induced lactic acidosis has not been studied. The efficacy of ongoing sodium bicarbonate in lactic acidosis, without renal replacement therapy, is unclear. In a retrospective study, solitary sodium bicarbonate administration in patients with lactic acidosis was associated with increased mortality [9]. They concluded that sodium bicarbonate administration alone, without inhibiting lactate production or enhancing lactate clearance, could negatively impact survival in patients with lactic acidosis. In our case, our patient’s lactic acidosis was initially responsive to a sodium bicarbonate infusion. However, the patient later developed tumor lysis syndrome after initiation of prednisone, which worsened her renal function, leading to progressive metabolic acidosis that became unresponsive to sodium bicarbonate, requiring CVVH. The impact of CVVH on mortality in the setting of malignancy-induced lactic acidosis is controversial. It has been shown that in patients with metformin-associated lactic acidosis requiring ICU-level care, the mortality rates were similar between the hemodialysis and non-hemodialysis groups (31.3% versus 28.6%, not statistically different), despite the hemodialysis group having higher illness severity scores [10]. In the dialysis group, the mean pH was 7.11 with CO_2_ 9.8 mmol/L and lactate 11.2 units/L. In a case series, early treatment with CVVH in metformin-associated lactic acidosis patients in shock showed favorable outcomes [11].

The definitive treatment of lactic acidosis is reversal of the underlying etiology. Thus, chemotherapy is the treatment of choice in the case of a malignancy-induced lactic acidosis. However, the initiation of chemotherapy may be limited by a patient’s clinical status, specifically renal and hepatic functions, as clearance of chemotherapy relies on these systems. Thus, the timing of bicarbonate and CVVH initiation is important to maintain electrolyte balance and renal function, allowing for the safe initiation of chemotherapy. In this case, chemotherapy was not dose reduced (the exception being vincristine dose reduction due to the patient’s age) on the basis of the relative risk–benefit tradeoff of early organ dysfunction weighed against the need for aggressive lymphoma management.

Double expressor DLBCL is an aggressive lymphoma. In double expressor lymphoma, MYC (MYC proto-oncogene, BHLH transcription factor) and BCL2 (B-cell leukemia/lymphoma 2) are overexpressed at protein levels (not at the gene level, as in the case of double hit lymphoma), as identified by immunohistochemical testing. Per the World Health Organization (WHO), greater than 40% MYC overexpressing cells and greater than 50% BCL2 overexpressing cells must be present to be diagnosed with “double expressor” [12]. Double-expressor DLBCL confers a poor prognosis overall. Due to the aggressive nature of double-hit or double-expressor DLBCL, it is recommended to enroll patients in clinical trials, if available, as standard R-CHOP induction is associated with loss of long-term disease control in about one-third of patients [13]. However, in our patient, the severity of metabolic derangements and deteriorating clinical course inherent in the Warburg effect warranted urgent initiation of therapy before specific details of the DLBCL could be known. Our patient showed clear improvement in her clinical status after first starting chemotherapy and was recovering well; however, when she was nearing her next cycle of chemotherapy, her clinical status deteriorated again and her care had to be re-escalated to the ICU. Ultimately, the early benefits of timely bicarbonate infusion, CVVH, and chemotherapy, arguably successful in this patient, were offset by evolving infection and development of sepsis, with progressive liver failure.

## Conclusions

The Warburg effect is an uncommon, poorly understood, and challenging clinical scenario, requiring prompt recognition and treatment. This entity should be suspected in patients with hematologic malignancies who develop both type B lactic acidosis and hypoglycemia. Early initiation of chemotherapy, along with aggressive supportive measures to correct metabolic abnormalities and treat the underlying process, are keys to the management of the Warburg effect. Despite prompt initiation of chemotherapy, the Warburg effect remains a significant negative prognostic indicator in the setting of malignancy. Further studies are needed to clarify the role of hemodialysis in this setting.

## Data Availability

All data generated or analyzed during this study are included in this published article.

## Abbreviations

DLBCL: Diffuse large B cell lymphoma
CVVH: Continuous veno-venous hemofiltration
R-CHOP: Rituximab, cyclophosphamide, doxorubicin, vincristine, prednisone
MYC: MYC proto-oncogene
BCL2: B-cell leukemia/lymphoma 2
CNS: Central nervous system
GCB: Germinal center B cell
MUC1: Mucin 1
